# Comparison of Eye Axial Length Measurements Taken Using Partial Coherence Interferometry and OCT Biometry

**DOI:** 10.3390/vision8030046

**Published:** 2024-07-26

**Authors:** Nicola Rizzieri, Alessio Facchin

**Affiliations:** 1 Optometry and Vision Science, University of Latvia, LV-1586 Riga, Latvia; nirizzieri@gmail.com; 2 Rizzieri Vision Center, 46041 Asola, Italy; 3 Neuroscience Research Center, Department of Medical and Surgical Sciences, Magna Graecia University, 88100 Catanzaro, Italy; 4 Institute of Research and Studies in Optics and Optometry, 50059 Vinci, Italy

**Keywords:** axial length, biometry, myopia, partial coherence interferometry, OCT

## Abstract

This study evaluates the inter-device measurement properties of partial coherence interferometry (PCI) and spectral domain optical coherence tomography (SD-OCT) in measuring axial length, particularly for myopia management. We recruited 82 eyes from 41 adult participants with a mean age of 31.0 ± 17.6 years and a mean spherical equivalent of −2.20 ± 2.28 D. Axial length was measured using SD-OCT and PCI for both the right and left eyes. Agreement between the two measurements was assessed using Bland–Altman analysis, and graphs and values were compared with linear mixed models. The results show a near-to-zero and non-significant bias between measurements. The 95% limits of agreement showed a value of 0.06 mm. Both devices can accurately measure the axial length. OCT biometry performed with SD-OCT can be successfully interchanged with partial coherence interferometry, but they should be cautiously interchanged when performing longitudinal comparisons.

## 1. Introduction

Ocular biometry is a fundamental procedure in designing intraocular lenses and anterior segment surgeries that involves the analysis of the eye structures along the visual axis [1,2]. In recent years, this technique has become an increasingly important tool for monitoring myopia progression. As a matter of fact, in the case of myopia, the axial length (AL) is significantly more sensitive in determining progression than optical refraction. AL should be the newest gold standard for assessing myopia progression, high myopia, and the risk of visual impairments [3]. The measurement of AL over time provides valuable insight into the risk of developing myopia and its progression. Furthermore, it enables more effective myopia control strategies. Recently, biometric instruments have been explicitly designed to clinically manage myopia [4].

AL measurement must be precise, accurate, and reliable in order to ensure quality and long-term healthcare. Therefore, it is crucial to measure AL using reliable technology. Moreover, multiple measurements are essential to guaranteeing accuracy.

Different techniques, including ultrasound, interferometry, and optical coherence tomography, can be used to assess axial length [5]. Some studies have compared the measurement properties of these different instruments. Pedersen et al. [6] compared partial coherence interferometry (PCI) with a swept-source optical coherence tomography (SS-OCT) device using 74 subjects. They found no statistically significant difference in the mean AL measurements (0.004 mm). In 2019, Sikorski and Suchon [7] presented a new universal method for AL measurement, usually referred to as OCT biometry (B-OCT), which is implemented in a commercially available spectral domain, OCT (SD-OCT), and potentially possible in other OCT devices in the future. They compared the new B-OCT method with the well-established SS-OCT method using 164 healthy eyes. They found that both devices can successfully measure AL and other ocular parameters, with the SS-OCT method having a lower failure rate in cataract patients (68%). The mean AL measurement difference in healthy eyes was −0.001 mm, and the 95% limits of agreement (LoAs) on the measurement difference ranged from −0.034 to 0.031. These results show that both devices performed almost identically. Kanclerz et al. [8] compared an updated version of B-OCT with SS-OCT and an optical low coherence reflectometer (OLCR) in a study with 63 participants. The difference in axial length measurement was statistically significant after the post hoc test (*p* < 0.001) between the B-OCT and SS-OCT devices, but not clinically relevant (less than 0.01 mm). The difference between B-OCT and OLCR was not statistically significant. Therefore, the authors consider the updated version of the B-OCT method to be interchangeable with the two others optical biometer modalities. Considering the previous results, this study aims to compare the AL measurement capabilities of two recent optical, no-contact instruments based on two distinct technologies: partial coherence interferometry (PCI) and optical biometry based on SD-OCT (B-OCT). This study aims to evaluate the possibility of using the two devices interchangeably in both clinical and research settings involving ocular biometry. We evaluated them in terms of the concordance and agreement of measurements.

## 2. Materials and Methods

### 2.1. Subjects

We primarily performed a power analysis to assess the minimum sample size required to detect an effect. Considering a paired t-test with a mean difference of 0.01 mm, SD = 0.03, alpha = 0.05, and power = 0.80, a minimum sample of 73 units is required. A total of 82 eyes of 41 participants, randomly selected from an optometry and contact lens office, were used in the study. The mean age was 31.0 ± 17.6 years (range 10–73, 26 females, 15 males), and the mean spherical equivalent was −2.20 ± 2.28 D. The extensive age range of the sample reflects the actual circumstances of a real-life clinic including both children and adults. Therefore, the instruments can be used on various patients. This helps to avoid issues related to studies involving only children, such as poor compliance, movement, and fixation problems. Adapting an instrument for myopia progression does not necessarily imply that it cannot be used for other purposes. The exclusion criteria were as follows: previous ocular surgery; refractive surgery; eye trauma; known corneal, lens, and macular disease. Participants or their guardians signed an informed consent form before participating. The study was conducted according to the principles of the Declaration of Helsinki and approved by the institutional review board of the Institute for Research and Study in Optics and Optometry (IRSOO, Vinci, Italy).

### 2.2. Instruments

The Oculus Myopia Master^®^ (Oculus Optikgeräte; Wetzlar, Germany; partial coherence interferometry, PCI; software version 1.4 r.1) is a specifically designed optical biometer for myopia management. It simultaneously measures axial length, corneal curvature, and objective refraction, saving valuable time. The pupil size, horizontal visible iris diameter, and an AL percentile growth chart are also displayed. For this research, we collected six consecutive AL measurements for each eye of every participant during a single acquisition session. The Optopol REVO FC 80^®^ (Optopol Technology; Zawiercie, Poland; spectral domain optical coherence tomography, SD-OCT; biometry module, software version 11.5.0) is an anterior and posterior segment OCT with a dedicated module for axial length measurement (B-OCT). We collected the ten vertical and horizontal measurements in a single acquisition session for each eye of every subject.

### 2.3. Procedure

The same well-trained examiner (N.R.) performed all measurements in a dimly lit (15 lx) room in an optometric office. We included both eyes of each subject in the evaluation and collected all the biometric measurements. We measured all eyes (*n* = 82) with both the PCI and B-OCT devices in a balanced random order. Each eye and participant underwent a specific sequence of measurements (acquisition). Before the subjective refraction, the examiner performed a non-cycloplegic autorefraction with the PCI device. All participants underwent a non-cycloplegic distance subjective refraction. The final point of refraction was the maximum positive sphere for maximum visual acuity. For this study, we used one specific sequence of measurement for both instruments, which included six measurements from PCI and ten from B-OCT—this permits the application of a more sophisticated analysis model.

### 2.4. Statistical Analysis

We performed a power analysis using the G*Power software (version 3.1) to determine the minimum sample size needed to detect a significant effect [9]. We reported the descriptive statistics using mean and standard deviation for continuous variables, and frequency for nominal variables. During automatic acquisition, each instrument may lose some measurements. For this reason, we were not able to collect all participants’ 6 trials for PCI and 10 trials for OCT. We documented the rate of missing data points. The correlation between the mean measurements was performed using the Concordance Correlation Coefficient (CCC).

We compared the AL measurements using a linear mixed model (LMM) with the within-fixed factors Instruments and Measurement and the random factor Eye. This model allows for the assessment of the differences between measurements, and is more robust in case of missing data points (the outliers which are automatically removed by the instruments). We performed agreement analysis using Bland–Altman analysis (BA) and plotted the mean measurement value as a single output of the instrument. We compared the mean value using a paired sample t-test. We defined the 95% limits of agreement as the 1.96 × standard deviation of the difference between the two measurement techniques. We applied a regression model to the BA data to assess the trend differences. The relationship between the difference in measurements and the age of each participant was evaluated using Pearson correlation. Finally, the within-session test–retest repeatability was assessed between measurements, considering the missing data, separately for each instrument using intraclass correlation.

## 3. Results

Table 1 summarises the descriptive statistics of the participants.

Regarding the data points lost during a single acquisition, we followed an accurate approach. For PCI, we noted missing measurements in a significant portion of the participants, obtaining all six measurements only for seven eyes (8.5%). The maximum number of missing measurements was three, with a mean of 1.77 ± 0.77. For B-OCT, we observed a range of missing measurements between zero and five for ten measurements. However, 58 acquisitions (70.7%) presented all ten measurements, with the mean of lost data being 0.60 ± 1.18. This comprehensive data collection process should instil confidence in the robustness of our research.

We then performed a correlation between the mean measurements of the two instruments. This correlation was very high, at CCC = 0.9995 (95% CI 0.9993 to 0.9997). This result was compared to those obtained in animal studies [10] (ICC = 0.92), presenting a significantly higher value (*p* < 0.001).

The difference in measurement between instruments was not significant for the factor Instrument [F_(1,1020)_ = 1.07 *p* = 0.30], for the factor Measurement [F_(1,1020)_ = 1.80 *p* = 0.18], and for their interaction [F_(1,1020)_ = 1.00 *p* = 0.31]. This confirms the equivalence of the two technologies and the lack of difference between their measurements.

From a clinical point of view, the mean value of each instrument is more advantageous than each single value. A comparison of the mean value given for each eye using a paired sample *t*-test showed no significant difference (t(80) = 0.5, *p* = 0.56) in this case. The descriptive values are reported in Table 1.

**Figure 1:**
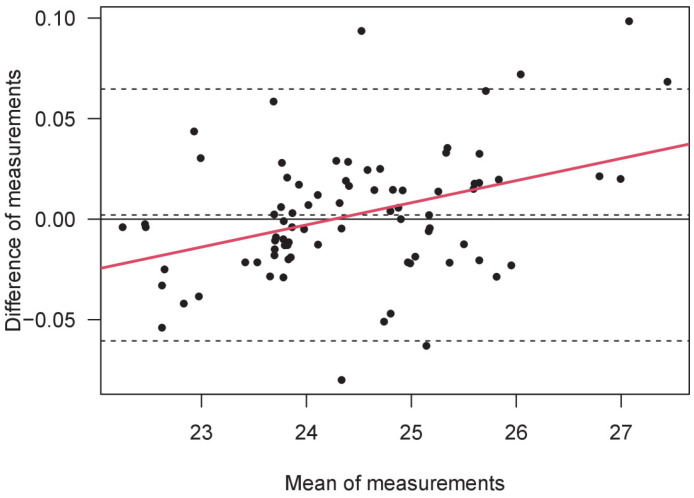
A Bland–Altman plot of the measurements of axial length (mm) obtained by B-OCT and PCI. The AL mean value is shown in the abscissa, while the difference between AL measurements is shown in the ordinate (B-OCT minus PCI). From top to bottom, the three horizontal dashed lines denote the upper limit of agreement (LoA), the mean bias, and the lower limit of agreement. Linear regression is represented by the continuous red line.

The Bland–Altman analysis showed a mean difference (bias) of 0.00205 mm, with an upper limit of 0.065, a lower limit of -0.061mm, and a total difference of 0.062 mm. Linear regression of the BA data showed a significant value (Beta = 0.38, *p* < 0.0005, R^2^ = 0.15), revealing a high difference between short and long eyes in two opposite directions (Figure 1). Finally, we assessed the correlation between the difference in measurements and the age of each participant. The result is not significant. The within-session test–retest repeatability was higher and equal for both instruments, with a value of ICC = 0.999.

## 4. Discussion

This study compares two recent optical, no-contact methods to measure the axial length of the eye: partial coherence interferometry (PCI) and optical biometry from spectral domain optical coherence tomography (B-OCT). AL measurements are crucial not only to the calculation of IOLs, but also to the distinction between axial and curvature refractive errors. Moreover, AL is becoming the new gold standard for monitoring myopia progression in young patients and the efficacy of management strategies [11].

The inclusion of adult participants and a wide range of AL beyond those usually considered for myopia progression was motivated by two principles from both a methodological and measurement perspective. By using adult participants only, we excluded all child-related problems in practical measurement, such as poor compliance, tolerability, movement, attention, and fixation problems, and this allowed us focus specifically on the measurement. Secondly, the AL measurement could be assessed independently from refraction and myopia management; as a result, we found that the instruments should be interchangeable regardless of refraction status and scope. Nevertheless, studying AL in myopia progression is an interesting topic, especially considering hyperopic subjects, for a better understanding of the roles of peripheral refraction and superimposed retinal defocus [12]. Hyperopic refraction is a risk factor for myopia development when it is lower than the minimum value expected for a specific age range. This condition, known as pre-myopia, is associated with a significantly higher risk of developing myopia, and can occur up to four years before myopia onset in pre-myopia subjects compared to their age-matched counterparts, who remain emmetropes [13]. AL measurements, in this case, may indicate that the patient has lower hyperopic refraction than average, with a greater axial length and flatter corneal curvature, enabling the eye care practitioner to manage the condition early on.

Regarding the missing data points, OCT technology has proven to be more reliable in terms of the number of true measurements effectively taken during each acquisition. This procedure is automatic for each instrument and transparent for the examiner. However, it is recommended to check the number of measurements, as many measurements could be lost, particularly with PCI technology.

The obtained results first showed that there is no statistical mean difference between the two instruments. For a better investigation and comparison, we took every instrument measurement within a single acquisition session using a more sophisticated model of analysis (LMM). Even with a proper sample size and power, the differences between instruments were not significant. Also, the studies of other authors support the absence of a mean difference with similar results, or have found a statistically significant outcome that proved to be clinically irrelevant [6,7,8,11,12,14]. In addition, the within-session test–retest repeatability (correlation) was very high, as expected. According to the factor measurement in LMM, there are no mean differences (bias) in the measurements within each acquisition. It is only necessary to pay attention to the number of valid measurements that have been recorded by the instrument, as previously mentioned. The within-session measurement is high and no specific problems emerged in this area.

The 95% LoAs between instruments were between −0.061 and +0.065 mm; this is the most crucial result to consider. Furthermore, the maximum difference was more significant for smaller and longer AL values, as indicated by the regression in BA values. In other words, the two instruments work differently—not in absolute terms, but more so when small and large eyes are considered. This highlights the importance of considering these differences when conducting research on AL measurement.

Mattern et al. [11] compared different optical biometers to the SS-OCT biometer. The LoAs between the two devices were from −0.06 to +0.08 mm. The PCI offers higher agreement and consistency compared to well-known PCI and SS-OCT devices [14]: the LoAs between the three devices were from −0.07 to no more than 0.12 mm. The PCI biometer used in the current study was recently compared with an optical low-coherence reflectometry (OLCR) method in a study involving children and adults [14], showing an LoA of −0.16 to +0.08, with better agreement in the adult group. The authors suggest caution when using biometers interchangeably. Kanclerz et al. [8] investigated the AL measured by the B-OCT device and two validated devices, one SS-OCT and one OLCR. The LoAs were −0.03 to +0.05 mm (B-OCT and SS-OCT) and −0.05 to 0.04 mm (B-OCT and OLCR). The authors suggest that they can be used interchangeably.

The LoA of about +/− 0.06 mm obtained in the current study is comparable to those found in the studies mentioned above, where the authors concluded that PCI and B-OCT devices could be used in a clinical setting, but cautiously when interchanged—for example, in multicentric studies and eye clinics/centers with different instruments. We demonstrated that the two instruments used in this study can be adopted for young and adult patients and perform well for a wide range of eye axial lengths. In any case, clinicians must pay attention when measuring high hyperopic and high myopic patients. In their study, Garcia Ardoy et al. [15] showed that SS-OCT could accurately measure longer eyes, steeper corneas, and larger horizontal visible iris diameters compared to a PCI-based device. This difference may be caused by the technical features of the technology of the two devices, with SS-OCT being recognized as one of the best optical biometers currently available, performing better than PCI and OLCR devices [16,17].

Moreover, anatomical differences between average-length and long eyes may affect the measurement results [18]. Specifically, the position of the fovea, or the best eccentric fixation, may be difficult to find with PCI compared to SS-OCT devices. Even the density of the lens or changes in the refractive indices of the ocular media, which are associated with age or refractive errors, could be potential sources of error for different device technologies [19,20]. In these two scenarios, it would be better to use the same device for longitudinal AL measurement [21,22].

Considering the studied population, the application of these results specifically concerns the follow-up management of myopia and refractive surgery in adulthood. The two main results—the LoA of ±0.06 mm and larger differences for longer (and shorter) eyes—also have profound implications in this field. Myopia and refractive surgery need to be regularly checked for years, and it is easy to use different instruments based on different technologies during all follow-up examinations. The key findings found here should be taken into consideration when drawing conclusions from monitoring over years. Additionally, maintaining comprehensive patient records that include all measurements and device details will aid in accurate long-term monitoring and assessment.

Finally, the possible correlation between age and the difference in axial length measurement was assessed. Our findings, which reveal that age has no significant relationship with the difference in measurement, have the potential to inspire future studies in the field of myopia management. We anticipate that these results will be applicable to the overall population, regardless of the age of the participants, and will spark new research directions and clinical applications.

One limitation of this study is that it primarily included adult participants. Future studies could apply the same methods only to younger populations who are involved in myopia management, or assess the between-session repeatability of these methods over weeks, also considering specific populations such as refractive surgery and ortho-K patients, keeping in mind their follow-up management.

The future direction of instrument comparison has to consider all methods of AL measurement, even those not included in the current research, such as OLCR and SS-OCT. Focusing on the repeatability of a single technology and the comparison between technologies will be crucial.

From a clinical perspective, both systems can be adopted in both pediatric and adult populations, due to their high precision and consistency. Both devices can be used for myopia management purposes. The B-OCT method is suitable for myopia control follow-up visits due to its patient-friendly configuration, high accuracy, and ease of use. However, it is crucial to exercise caution when using the devices interchangeably, especially for very long and very short eyes. We recommend taking multiple measurements and always using the average result with standard deviation, to ensure the highest level of accuracy and patient safety.

## Figures and Tables

**Table 1:** Descriptive characteristics of the samples collected.

	n	Mean ± SD	Range
Age	41	31.0 ± 17.6	9 to 72
Sex	41	26F/15M	
Refraction sphere (D)	82	−1.95 ± 2.26	−6.50 to +3.25
Refraction CYL (D)	82	−0.47 ± 0.69	−3.25 to 0.00
Refraction SE (D)	82	−2.18 ± 2.27	−7.25 to 2.875
Axial length B-OCT (mm)	82	24.45 ± 1.11	22.25 to 27.48
Axial length PCI (mm)	82	24.45 ± 1.09	22.25 to 27.41
Axial length difference (B-OCT-PCI, mm)	82	0.0021 ± 0.0320	−0.0801 to 0.0984

Note: CYL = cylinder; SE = spherical equivalent; PCI = partial coherence interferometry; B-OCT= OCT biometry; SD = standard deviation.

## Data Availability

The data presented in this study are available on request from the corresponding author. The data are not publicly available due to restrictions included in the informed consent provided by participants.
